# A new method for sampling African swine fever virus genome and its inactivation in environmental samples

**DOI:** 10.1038/s41598-021-00552-8

**Published:** 2021-11-03

**Authors:** Aleksandra Kosowska, Jose A. Barasona, Sandra Barroso-Arévalo, Belén Rivera, Lucas Domínguez, Jose M. Sánchez-Vizcaíno

**Affiliations:** 1 VISAVET Health Surveillance Center, Complutense University of Madrid, Madrid, Spain; 2 Department of Animal Health, Faculty of Veterinary, Complutense University of Madrid, Madrid, Spain

**Keywords:** Microbiology, Molecular biology, Diseases, Risk factors

## Abstract

African swine fever (ASF) is currently the most dangerous disease for the global pig industry, causing huge economic losses, due to the lack of effective vaccine or treatment. Only the early detection of ASF virus (ASFV) and proper biosecurity measures are effective to reduce the viral expansion. One of the most widely recognized risks as regards the introduction ASFV into a country is infected animals and contaminated livestock vehicles. In order to improve ASF surveillance, we have assessed the capacity for the detection and inactivation of ASFV genome by using Dry-Sponges (3 M) pre-hydrated with a new surfactant liquid. We sampled different surfaces in ASFV-contaminated facilities, including animal skins, and the results were compared to those obtained using a traditional sampling method. The surfactant liquid successfully inactivated the virus, while ASFV DNA was well preserved for the detection. This is an effective method to systematically recover ASFV DNA from different surfaces and skin, which has a key applied relevance in surveillance of vehicles transporting live animals and greatly improves animal welfare. This method provides an important basis for the detection of ASFV genome that can be assessed without the biosafety requirements of a BSL-3 laboratory at least in ASF-affected countries, which may substantially speed up the early detection of the pathogen.

## Introduction

African swine fever (ASF) is one of the most relevant swine diseases owing to its sanitary significance and socioeconomic consequences for a considerable number of countries^1^. ASF has, in the last few decades, shown a remarkable capacity for transboundary and transcontinental spread, with a growing number of outbreaks of the disease on five different continents and in more than 50 countries^2^. Countries affected by ASF struggle to control and minimize losses, while countries that are still ASF free confront an increased risk of pathogen introduction^3^.

ASF is caused by infection with a large, enveloped double-stranded DNA virus, the only member of the *Asfaviridae* family^4^. There is, as yet, no commercial vaccine or effective treatment available to protect against the disease signifying that the principal tool for disease control is preventive measures^1^. With the ongoing process of globalization, viral introduction into a country is primarily facilitated by infected animals and their products, which may be transported over long distances from infected countries^5,6^. Other sources of infection are related to contaminated fomites, including livestock vehicles, feed, or the clothing and shoes of those working with animals^7^.

Vehicles transporting pigs to farms, markets, or slaughterhouses, delivering feed, or collecting carcasses represent a great risk for disease transmission^8^. The role of contaminated vehicles has been evaluated in several studies, which have concluded that returning trucks are the highest risk for ASF introduction into the European Union (EU) when compared with other transport-associated routes^8,9^. As the level of disinfection of vehicles is an important parameter for the risk assessment of ASF environmental control, pathogen analysis at disinfection points would be advisable in order to prevent possible viral transmission between pig farms^6^.

The prevention and control of the ASF are, at present, based principally on the early detection of the disease through timely recognition in the field and efficient laboratory diagnosis^10,11^. In this respect, a good surveillance program, the availability of facilities and resources, and the preparedness of veterinary services are determinant factors, since samples potentially infected with ASF virus (ASFV) should be handled in a laboratory with an appropriate level of bio-contamination, at least in ASF-free countries, that is not widely available. An inactivated virus could, however, be analyzed in Biosafety level 2 (BSL-2) laboratories. The use of a new method that would ensure the complete inactivation of the virus but also allow the preservation of the viral genetic material would, therefore, be of great interest as regards facilitating and accelerating ASFV diagnosis, particularly at critical control points such as vehicle disinfection and animal transportation.

Here, we assess the effectiveness of a new sampling method based on Dry Sponges (3 M Dry-Sponge; 3 M, Madrid, Spain) pre-hydrated with a new surfactant and virus-inactivating liquid for pathogen nucleic acid detection employing quantitative PCR (qPCR). We hypothesized that this safe and simple sampling method could be potentially useful for the control of livestock vehicles and the effective surveillance of ASF. In order to take effective action for the rapid identification and further control of ASF, the specific objectives of this work are: (i) to evaluate the capacity of Dry Sponges (3 M) pre-hydrated with surfactant and virus-inactivating liquid to detect ASFV DNA in the environment, including animal skins, and (ii) to validate the virus inactivating properties of the surfactant liquid in an in vivo experiment.

## Material and methods

Animal trials were conducted in the Biosafety level 3 (BSL-3) animal facilities at the VISAVET Centre, at the University Complutense of Madrid. All the animals were individually ear-tagged and acclimated for one week before the experiments began. Access to water and food was provided ad libitum throughout the studies. Animal care and procedures were performed in accordance with the guidelines of good experimental practices, following European, national and regional regulations, and were approved by the Ethics Committee of the Comunidad de Madrid (reference PROEX 159/19). Guidelines for the ARRIVE 2.0 for the care and use of laboratory animals were also followed.

### Study design

Two independent kinds of samplings were carried out to evaluate the efficacy and inactivation capacity of the Dry Sponges (3 M) pre-hydrated with surfactant liquid and the results were compared to those obtained using a traditional sampling method with a cotton swabs.

### Experiment 1: The efficacy of Dry Sponge (3 M) for ASFV genome detection

To evaluate the efficacy of the sponges as regards preserving the ASFV genome and allowing viral detection, a paired environmental sampling of an ASFV-contaminated environment (Supplementary material S1) and animal skins was performed as part of an experimental study. In this study, five female wild boar (*Sus scrofa*) were experimentally infected with the ASFV Armenia07 (Arm07) isolate through intramuscular inoculation with 10 50% hemadsorption dose (HAD_50_). Necropsy was performed on all the animals, a total of 21 tissue samples (lymph nodes, spleen, liver, lung, heart, kidney, brain, urinary bladder, intestine, diaphragm, bone marrow, synovial membranes and meat-juice) were tested for the presence of ASFV by employing qPCRs.

During the experimental period, the facilities were treated three times per week, using Virkon™ S broad-spectrum disinfectant (LANXESS, Suffolk, UK). For safety reasons, the pen equipment and the places where the animals lay to sleep or rest were cleaned only with water. No additional cleaning was performed following the termination of this study when the various surfaces were sampled using the Dry Sponges (3 M) and conventional swabs so as to compare the sensitivity of both methods.

To evaluate the capacity of viral inactivation of the new sampling method, another experiment was carried out using live animals, as described below (Experiment 2).

#### Sampling material

The environmental sampling was carried out using Dry Sponges 3 M (3 M Dry-Sponge; 3 M, Madrid, Spain). This biocid-free cellulose sponge comes with a sample bag that ensures safe transportation to the laboratory without compromising the sample. It is commonly used in food industry to identify potential food-borne hazards (manufacturer's website). The Dry Sponge (3 M) can be pre-hydrated with a wide range buffers and enrichment media to meet the sampling needs.

In this work, the sponges were pre-hydrated with isotonic surfactant liquid (Spanish patent n° P2115ES00) obtained by the mixing of equal parts of two previously prepared solutions. Solution A was composed of isopropyl alcohol 99.8%, ethanol 99.8%, methanol 99.9% and glycerol. On the other side, Solution B was composed of disodium phosphate, sodium dodecyl sulfate 0.1% (SDS) and nuclease-free water. Alcohols are commonly used for disinfection purposes due to their broad activity against bacteria, viruses, and fungi^21^. They are amphiphilic compounds, as they possess both hydrophilic and lipophilic (hydrophobic) properties, leading to lipid membrane dissolution and protein denaturation. In addition, the presence of polar oxygen atoms weaken the lipophilic interactions between the non-polar residues and increase the internal affinity of the membrane for water, thus destabilizing the protein structure^21–23^. ASFV belongs to the enveloped variant vulnerable to disinfectants interrupting its lipid-envelope structure^24^. In addition, the surfactant liquid also contains SDS, the ionic detergent class of proteins denaturants, used routinely in the laboratory for estimation of molecular weight of proteins^25^.

#### Environmental sampling

We selected six different sites in direct contact with the animals (feeders, troughs, and floor) and two non-contact sites (facilities: walls, doors and metal bars). During environmental sampling, we also included samples collected from the skin of four animals at the end of the study. Two sponges were employed for each sampling site and were gently rubbed over the surfaces, while one sponge was employed per animal and was gently rubbed over the left scapula and flank regions. The sampling from the animal´s skin was carried out before the necropsy, to avoid the contamination with blood. These sponges were pre-hydrated with 15 ml of isotonic surfactant liquid that makes it possible to collect nucleic acids from surfaces and other substrates, as previously described by Martínez-Guijosa et al*.* ^12^*,* and Fernández-de-Mera et al*.,*^13^. The same environmental sites, with the exception of the animal skins, had previously been sampled for the same contiguous surface area with cotton swabs (Deltalab, Barcelona, Spain) pre-hydrated with 1 ml of phosphate-buffered saline (PBS) 1X. All the samples were processed immediately after collection and stored at − 80 °C until further use.

#### Laboratory procedures

In the laboratory, 1.5 ml of retained fluid was extracted from each sponge sample, while the swabs were directly dipped in 800 µl of PBS 1X. All the samples were collected in tubes and investigators responsible for laboratory procedures were blinded to treatment group allocation. Viral DNA was extracted from 200 µl of solution taken from the bottom of the tube using the High Pure Template Preparation Mix Kit (Roche Diagnostics GmbH, Mannheim, Germany) according to the manufacturer’s instructions. Positive and negative controls were used in DNA extraction. The detection of ASFV DNA was performed using the Universal Probe Library (UPL) real-time PCR previously described by Fernández-Pinero et al*.*^14^. The OIE recommended this real-time PCR method for ASF diagnosis based on the VP72 DNA sequence detection, using the primers (forward 5′-CCCAGGRGATAAAATGACTG-3′; reverse 5′-CACTRGTTCCCTCCACCGATA-3′) and UPL#162 probe (5′-FAM-GGCCAGGA-TAMRA-3′) (Roche cat no. 04694490001). All qPCR reactions were carried out in CFX Connect™ Real-Time PCR Detection System (BioRad, Berkeley, USA) according to the following cycling conditions: 95 °C for 5 min, followed by 45 2-step cycles at 95 °C for 10 s and 60 °C for 30 s. Samples were tested in duplicate and an average of the copies/µl was considered. A positive qPCR result was determined by identifying the threshold cycle value (Ct) at which reporter dye emission appeared above the background within 40 cycles. Nuclease-free sterile water was used as a negative control. The presence of ASFV genome in environmental positive samples was confirmed by sequencing the 3´end of the VP72 coding sequence, which differentiates up to 24 distinct genotypes^15^.

Viral load of positive samples was determined by employing absolute quantification based on a standard curve constructed using serial tenfold dilutions of a plasmid in triplicate of known amounts of Kit TOPO™ TA Cloning™ (Invitrogen™) containing a specific fragment of ASFV DNA. A standard curve was fitted with lines showing correlation coefficients of 0.99 (data not shown). Viral loads were expressed in absolute terms of DNA equivalents per microliter (genome copies/μl). The limit of detection was 10 copies/µl.

### Experiment 2: Validation of inactivating properties of the surfactant liquid (in vivo experiment)

#### Animals

Ten 3 month-old castrated male Landrace breed pigs weighing 20–25 kg, were obtained from an authorized breeding center in Segovia, Spain. The pigs were randomly divided into four different groups, three of which were intramuscularly inoculated with 1 ml injections of inoculum (specified below) in the left semimembranosus muscle (Table 1). Local infiltration with lidocaine (Anesvet®, Ovejero, Spain) was used around the inoculation site to provide analgesia.

The first group of pigs was inoculated with the pool of environmental samples collected with Dry Sponges (3 M) from an ASFV-contaminated environment (Environment; n = 2). The second group was inoculated with a highly virulent hemadsorbing ASFV genotype II isolate, Armenia07 previously inactivated with the surfactant liquid (Armenia07; n = 2) and the third group was inoculated with the sterile surfactant liquid used to hydrate sponge (Control; n = 2). Four naïve pigs were kept in contact with and housed in the same pen as the inoculated animals (Naïve; n = 4). The random allocation of pigs to the treatment group was generated using random number generator (https://www.graphpad.com/quickcalcs/randomize1/).

Experiment 2 did not include a control group for Armenia07 due to the high risk of ASFV transmission between challenged and control pigs inoculated with the pool of the environmental samples. We have considered that the lethal effect (100% lethality) of the shedder-pig challenge exposure infection model at 10 HAD_50_ doses of ASFV Armenia07 has been widely described in pigs and wild boar in direct contact with infected animals and environments^26–30^.

#### Inoculum

Inoculum for the Environment group was prepared as a pool of environmental samples collected with the Dry Sponges (3 M). We selected four environmental samples: two samples with a low load of ASFV genome copies (Facilities A, Facilities B) and another two with a high load of ASFV genome copies (Feeder B, Floor A). We took 500 µl from each sample and added it to the tube. A total of 2 ml of inoculum was prepared, which was distributed to each animal on an equal basis (1 ml/animal).

The highly virulent Armenia07 isolate was used to prepare the inoculum for the second group (Armenia07). Viral titer was defined as the amount of virus causing hemadsorption in 50% of infected cultures (HAD_50_ per ml). Surfactant and virus-inactivating liquid was used as a thinner to perform serial dilutions of the virus. A high viral load of 10^5^ HAD_50_ was selected for the inoculum.

An inoculum with 1 ml/animal of the sterile surfactant liquid used for sponge hydration was prepared for the Control group.

**Table 1:** Protocol for each group of inoculated and control animals.

Group	Inoculum	Dose (genome copies/µl)
Enviroment (n = 2)	The pool of environmental samples	1.12 × 10^5^
Armenia07 (n = 2)	ASFV Armenia07 isolate	5.77 × 10^5^
Control (n = 2)	Surfactant liquid	–
Naïve (n = 4)	–	−

#### Clinical examination and euthanasia

Clinical signs, including rectal temperature, were recorded daily and expressed with a quantitative clinical score obtained by adding values for eight clinical signs, as described by Gallardo et al.^16,17^. These signs included fever (defined as a rectal temperature higher than 40 °C), anorexia, recumbence, skin hemorrhage or cyanosis, joint swelling, respiratory distress, ocular discharge and digestive findings. The body temperature was measured before each sampling and also, at 3, 6, 10, and 14 days post-inoculation (dpi). At the end of the experimental period (28 dpi), the animals were deeply anesthetized using intramuscular injections with tiletamine-zolazepam (Zoletil® 100 mg/ml, Virbac, France) and medetomidine (Medetor®, Virbac, France), then euthanized by employing intravenous injections of the T61® euthanizing agent (Intervet, Spain). Necropsy was performed on all the animals, and tissue samples (lymph nodes, spleen, liver, lung, heart, kidney, and bone marrow) were simultaneously collected for ASFV detection.

#### Sampling and ASFV DNA detection

EDTA blood and coagulated blood for the preparation of serum were collected from each animal on day 0 before inoculation and at 4, 7, 12, 17, 19, 24, and 28 dpi. The EDTA blood samples were processed immediately after their collection and serum samples were aliquoted and stored at -80 °C until further use. The DNA extraction and ASFV DNA detection from blood and tissues were performed using the same procedures previously described for environmental samples. Investigators responsible for the sampling of the environment and animals could not be blinded to the allocation group due to staff low number.

#### Antibody detection

Serum samples were analyzed for antibodies using a commercial ELISA based on the detection of the VP72 ASFV antigen (®Ingenasa-Ingezim PPA Compac K3; Ingenasa, Madrid, Spain) according to the manufacturer’s instructions.

#### Statistical analysis

The statistical analysis was conducted using SPSS 25 (IBM, Somar, USA). Fisher´s exact test was used to compare the results of the qPCR obtained from the two environmental sampling methods. The Mann–Whitney U test was used to compare the qPCR results between different surfaces in samples collected with the Dry Sponge (3 M). Significance was considered at a *p*-value < 0.05.

## Results

### Experiment 1: The efficacy of Dry Sponge (3 M) for ASFV genome detection

#### Clinical findings in an ASFV-contaminated environment

In the previous study, all the intramuscularly inoculated wild boar (n = 5) get infected with ASFV Arm07 isolate and developed clinical signs compatible with ASFV infection. The virus caused the rapid progression of the disease and the animals presented fever, depression and anorexia after viral inoculation. One of them had dyspnea and hemorrhagic diarrhea. At the moment of death or euthanasia, all the animals had high viraemia (1.37 × 10^8^ ± 0.96 × 10^8^ copies/µl). Before the necropsy, we have sampled animal´s skin/hair for subsequent analysis, presented below. A *post-mortem* analysis revealed pathological findings compatible with ASF, and the presence of ASFV DNA in tissues was confirmed in all the animals. These results have allowed us to confirm that an ASFV-contaminated environment was correctly established.

#### Capacity for viral detection in environmental samples

All the samples collected from surfaces confirmed the presence of ASFV DNA in the environment, and similar ASFV genome copy results were obtained using both environmental sampling methods (Table 2). There was no difference in ASFV DNA detection when sampling with Dry Sponges (3 M) or cotton swabs (Fisher´s exact Test; p > 0.05). Samples collected from surfaces in direct contact with the animals generally had a higher number of ASFV genome copies than those collected from surfaces that the animals could not directly access.

Samples collected from animal skins using Dry Sponges (3 M) contained a lower load of ASFV DNA (1.08 × 10^3^ ± 0.63 × 10^3^ copies/µl) than environmental surfaces (2.92 × 10^5^ ± 1.5 × 10^5^ copies/µl) (Mann–Whitney U test; Z = -2.71, *p* < 0.05).

**Table 2:** Presence of African swine fever virus (ASFV) in environmental samples.

Sampling site	qPCR results (genome copies/µl)	
	Dry sponge (3 M)	Cotton swab
Feeder A	1.55 × 10^5^	2.40 × 10^6^
Feeder B	1.21 × 10^6^	3.27 × 10^6^
Trough A	9.99 × 10^4^	6.10 × 10^2^
Trough B	2.13 × 10^5^	2.25 × 10^6^
Floor A	6.47 × 10^5^	6.43 × 10^5^
Floor B	1.87 × 10^3^	1.73 × 10^3^
Facilities A	2.17 × 10^3^	1.48 × 10^5^
Facilities B	4.86 × 10^3^	3.31 × 10^6^

### Experiment 2: Validation of virus inactivation properties of the surfactant liquid

#### Clinical findings and viral detection in inoculated and naïve pigs

All animals survived till the end of the study (28 dpi) and their data were included for analysis. Six pigs, previously divided into three groups (Environment, Armenia07 and Control), were inoculated with 1 ml of inoculum, and hosted jointly with other four animals (Naïve). The inoculated (n = 6) and naïve (n = 4) pigs did not develop any clinical signs of ASFV infection, and no viral DNA was detected in the blood samples obtained from these animals throughout the experimental period of 28 days. The body temperatures of the inoculated (39.3 °C ± 0.43) and naïve (39.4 °C ± 0.22) animals were within the normal range. Necropsy did not reveal macroscopic lesions compatible with ASFV infection, and ASFV DNA was not detected in any of the tissues analyzed.

#### Antibody detection

No anti-ASFV antibodies were detected in serum from any of the inoculated or naïve pigs.

## Discussion

To the best of our knowledge, this is the first study to evaluate a method for the detection and direct inactivation of ASFV in environmental samples and animal skin. The results obtained from two independent studies confirmed that the present method allows viral genome detection from different surfaces with a sensitivity similar to that of other commonly used methods (i.e., swabs), while simultaneously producing complete virus inactivation. The practical implications derived from these outcomes are numerous, since the new sampling method may streamline ASF diagnosis.

Environmental sampling obtained from Dry Sponge (3 M) samples had a similar ASFV load to that obtained from samples collected with a traditional method, using a cotton swab with PBS. As test sensitivity is maintained with the new sampling method, this could be indicative that ASFV DNA is well preserved despite viral inactivation and that it is possible to perform qPCR assays to detect ASFV DNA obtained from samples collected with these sponges. The Dry Sponge (3 M) will also allow sampling on much wider surfaces when compared to those that can be covered using swabs, thus increasing the chance of viral particle detection in contaminated animals and environments. This is particularly interesting in the case of livestock vehicles and transported animals, which are considered an important hazard for ASFV introduction^9^.

Another interesting property of the Dry Sponges (3 M) is their capacity to detect ASFV DNA on animal skins. This outcome was previously described by Martínez-Guijosa et al.^12^ in a study concerning *Mycobacterium tuberculosis* complex, during which the present methodology was used for pathogen monitoring in cattle. In our study, animal skin contained a lower amount of viral DNA than the other surfaces sampled. This could be explained by the fact that an animal´s skin/hair is a dynamic surface on which viral particles do not remain for long in comparison with other static surfaces. Pen facilities such as the floor, feeders or troughs can accumulate large amounts of the viral genome during long periods, owing to the stability of the ASFV. More knowledge regarding the survival of the infectious virus on animal skin and other environmental surfaces is, however, required.

We have additionally not only demonstrated that the present sampling method is of a sufficient sensitivity to detect viral DNA but have also confirmed the presence of the virus-inactivating properties. In our in vivo experiment, the inoculated animals did not become infected, despite being inoculated with a highly virulent isolate. We did not observe any ASF-compatible clinical signs or viraemia, and all the pigs remained in good health until the end of the experiment. All of these results have confirmed that the sampling method described in this work effectively inactivates the virus, thus providing a valuable tool for future ASF surveillance.

One of the most widely recognized risks of introducing ASFV is contaminated livestock vehicles. Previous epidemiological analyses of the outbreaks in Estonia (2015–2017) have demonstrated that the virus was most likely introduced by an indirect transmission pathway. On the majority of commercial farms, the virus was mainly introduced by contaminated fomites (vehicles, people, tools)^18^. Possible livestock vehicle contamination may originate from the excretions (feces, urine, oral/nasal fluid) of infected animals^19^. Proper cleaning and disinfection is, therefore, a crucial preventive action by which to avoid reinfection from environmental sources (SANTE/7113/2015: Strategic approach to the management of African Swine Fever for the EU). The implementation of this new sampling methodology may help to evaluate the presence of contamination at this critical point, thus contributing to the prevention and control of the disease.

The recent emergence of ASF has confirmed that improved non-invasive sampling techniques and better diagnostic tests for environmental samples are necessary for optimized disease control^20^. Since the absence of the residual ASFV DNA is a guarantee for the safety of live animals, the capacity of the Dry Sponge (3 M) for viral detection on animal skins could be particularly interesting in the case of transported pigs. This methodology could be employed as an additional tool in ASF diagnosis allowing the early detection of potential ASF infection in transported animals and greatly improving animal welfare. However, these properties would need further validation studies.

This methodology has been employed in recent studies, which have confirmed that this is a valuable tool with which to detect other pathogens such as SARS-CoV-2 on environmental surfaces^13^ and *Mycobacterium tuberculosis* complex on cattle skin^12^. It is, however, the first time that virus-inactivating properties of the surfactant liquid have been confirmed. If the virus is inactivated in the original sample, sample processing may be significantly accelerated. As DNA extraction and purification could be performed under BSL-2 laboratory conditions, many laboratories would have the ability to quickly diagnose the disease. The implementation of this new sampling methodology may help to increase the capacity for diagnosis at the main risk points for the disease, thus contributing to the prevention and control of the disease.

In conclusion, this is an effective method by which to systematically recover loads of ASFV genome from different surfaces, which has a key applied relevance as regards evaluating the effectiveness of disinfection in vehicles transporting live animals or products at risk of being contaminated. This method provides an important basis for the validation and early detection testing of ASFV that can be assessed without the biosafety requirements of a BSL-3 laboratory, at least in ASF-affected countries. In other words, this simple, rapid, and economic sampling method will reduce the true risk of ASFV transmission between farms, improve animal welfare, and avoid significant economic losses.

## Supplementary Information

Supplementary Legends.

Supplementary Video S1.

## References

[1] Arias M, Jurado C, Gallardo C, Fernández-Pinero J, Sánchez-Vizcaíno JM (2018). Gaps in African swine fever: Analysis and priorities. Transbound. Emerg. Dis..

[2] OIE. 2020 WAHID Database. https://www.oie.int/wahis_2/public/wahid.php/Diseaseinformation/Diseasedistributionmap. Accessed 18/01/2020.

[3] OIE. 2020. https://oiebulletin.com/?page_id=150. Accessed 09/10/2020.

[4] Dixon, L. K., Escribano, J. M., Martins, C., Rock, D. L., Salas, M. L., & Wilkinson, P. J. (2005). Asfarviridae. *Virus Taxonomy, Eighth Report of the ICTV*, 135–143.

[5] Bosch J (2017). Update on the risk of introduction of African swine fever by wild boar into disease-free european union countries. Transbound. Emerg. Dis..

[6] Beltrán-Alcrudo, D., Arias, M., Gallardo, C., Kramer, S. & Penrith, M. L. *African swine fever: detection and diagnosis—A manual for veterinarians. FAO Animal Production and Health Manual*. (2017).

[7] Sánchez-Vizcaíno JM, Mur L, Gomez-Villamandos JC, Carrasco L (2015). An update on the epidemiology and pathology of African swine fever. J. Comp. Pathol..

[8] Bellini S, Rutili D, Guberti V (2016). Preventive measures aimed at minimizing the risk of African swine fever virus spread in pig farming systems. Acta Vet. Scand..

[9] Mur, L., Martínez-López, B. & Manuel Sánchez-Vizcaíno, J. *Risk of African swine fever introduction into the European Union through transport-associated routes: returning trucks and waste from international ships and planes*. (2012). 10.1186/1746-6148-8-149 PMC3485109 22935221

[10] Gallardo MC (2015). African swine fever: A global view of the current challenge. Porc. Heal. Manag..

[11] Costard S (2009). African swine fever: How can global spread be prevented?. Philos. Trans. R. Soc. B Biol. Sci..

[12] Martínez-Guijosa, J. *et al.* Environmental DNA: A promising factor for tuberculosis risk assessment in multi-host settings. *PLoS ONE* **15**, (2020). 10.1371/journal.pone.0233837 PMC7259669 32470035

[13] Fernández-de-Mera, I. G. *et al.* Detection of environmental SARS-CoV-2 RNA in a high prevalence setting in Spain. *Transbound. Emerg. Dis.* (2020). 10.1111/tbed.13817 32894654

[14] Fernández-Pinero J (2013). Molecular diagnosis of african swine fever by a new real-time PCR using universal probe library. Transbound. Emerg. Dis..

[15] Quembo CJ, Jori F, Vosloo W, Heath L (2018). Genetic characterization of African swine fever virus isolates from soft ticks at the wildlife/domestic interface in Mozambique and identification of a novel genotype. Transbound. Emerg. Dis..

[16] Gallardo C (2015). Assessment of African swine fever diagnostic techniques as a response to the epidemic outbreaks in eastern european union countries: How to improve surveillance and control programs. J. Clin. Microbiol..

[17] Gallardo C (2015). Experimental transmission of African swine fever (ASF) low virulent isolate NH/P68 by surviving pigs. Transbound. Emerg. Dis..

[18] Nurmoja, I. *et al.* Epidemiological analysis of the 2015–2017 African swine fever outbreaks in Estonia. (2018). 10.1016/j.prevetmed.2018.10.001 30482617

[19] Davies K (2017). Survival of African swine fever virus in excretions from pigs experimentally infected with the Georgia 2007/1 Isolate. Transbound. Emerg. Dis..

[20] Blome, S., Franzke, K. & Beer, M. African swine fever—A review of current knowledge. *Virus Research* vol. 287 (2020). 10.1016/j.virusres.2020.198099 32755631

[21] Singh, D., Joshi, K., Samuel, A., Patra, J. & Mahindroo, N. Alcohol-based hand sanitizers as first line of defense against SARS CoV-2: A review of biology, chemistry and formulations. *Epidemiol. Infect.* (2020). 10.1017/S0950268820002319 PMC7550876 32988431

[22] Ingram LO (1976). Adaptation of membrane lipids to alcohols. J. Bacteriol..

[23] Golin AP, Choi D, Ghahary A (2020). Hand sanitizers: A review of ingredients, mechanisms of action, modes of delivery, and efficacy against coronaviruses. Am. J. Infect. Control.

[24] Juszkiewicz M, Walczak M, Mazur-Panasiuk N, Woźniakowski G (2020). Effectiveness of chemical compounds used against African swine fever virus in commercial available disinfectants. Pathogens.

[25] Bhuyan AK (2010). On the mechanism of SDS-induced protein denaturation. Biopolymers.

[26] Barasona JA (2019). First oral vaccination of eurasian wild boar against African swine fever virus genotype II. Front. Vet. Sci..

[27] Cadenas-Fernández E (2020). Adenovirus-vectored African swine fever virus antigens cocktail is not protective against virulent Arm07 Isolate in Eurasian wild boar. Pathogens.

[28] Rodríguez-Bertos A (2020). Clinical course and gross pathological findings in wild boar infected with a highly virulent strain of african swine fever virus genotype II. Pathogens.

[29] Sunwoo S-Y (2019). DNA-protein vaccination strategy does not protect from challenge with African swine fever virus armenia 2007 strain. Vaccines.

[30] Gallardo C (2018). African swine fever virus (ASFV) protection mediated by NH/P68 and NH/P68 recombinant live-attenuated viruses. Vaccine.

