# Supplementary material for: A new method for sampling African swine fever virus genome and its inactivation in environmental samples
Source: Sci Rep. 2021 Nov 3;11:21560. doi: 10.1038/s41598-021-00552-8 (PMC8566511; doi:10.1038/s41598-021-00552-8)
Supplement: Supplementary file 1 — Supplementary Legends. [file 41598_2021_552_MOESM1_ESM.docx]

Supplementary material S1. Video example of environmental sampling with the use of Dry Sponge (3M) pre-hydrated with the surfactant liquid.
